# *Helicobacter pylori* Induced Phosphatidylinositol-3-OH Kinase/mTOR Activation Increases Hypoxia Inducible Factor-1α to Promote Loss of Cyclin D1 and G0/G1 Cell Cycle Arrest in Human Gastric Cells

**DOI:** 10.3389/fcimb.2017.00092

**Published:** 2017-03-28

**Authors:** Jimena Canales, Manuel Valenzuela, Jimena Bravo, Paulina Cerda-Opazo, Carla Jorquera, Héctor Toledo, Denisse Bravo, Andrew F. G. Quest

**Affiliations:** ^1^Laboratorio de Comunicaciones Celulares, Facultad De Medicina, Centro de Estudios Moleculares De la Célula, Centro de Estudios Avanzados en Enfermedades Crónicas, Programa De Biología Celular y Molecular, Instituto de Ciencias Biomédicas, Universidad de ChileSantiago, Chile; ^2^Facultad de Ciencias de la Salud, Universidad Central de ChileSantiago, Chile; ^3^Laboratorio de Microbiología Molecular, Facultad de Medicina, Programa de Biología Celular y Molecular, Instituto de Ciencias Biomédicas, Universidad de ChileSantiago, Chile; ^4^Laboratorio de Microbiología Oral, Departamento de Patología y Medicina Oral, Facultad De Odontología, Universidad de ChileSantiago, Chile

**Keywords:** *Helicobacter pylori*, PI3K/mTOR, HIF-1α, Cyclin D1, cell cycle arrest

## Abstract

*Helicobacter pylori* (*H. pylori*) is a human gastric pathogen that has been linked to the development of several gastric pathologies, such as gastritis, peptic ulcer, and gastric cancer. In the gastric epithelium, the bacterium modifies many signaling pathways, resulting in contradictory responses that favor both proliferation and apoptosis. Consistent with such observations, *H. pylori* activates routes associated with cell cycle progression and cell cycle arrest. *H. pylori* infection also induces the hypoxia-induced factor HIF-1α, a transcription factor known to promote expression of genes that permit metabolic adaptation to the hypoxic environment in tumors and angiogenesis. Recently, however, also roles for HIF-1α in the repair of damaged DNA and inhibition of gene expression were described. Here, we investigated signaling pathways induced by *H. pylori* in gastric cells that favor HIF-1α expression and the consequences thereof in infected cells. Our results revealed that *H. pylori* promoted PI3K/mTOR-dependent HIF-1α induction, HIF-1α translocation to the nucleus, and activity as a transcription factor as evidenced using a reporter assay. Surprisingly, however, transcription of known HIF-1α effector genes evaluated by qPCR analysis, revealed either no change (LDHA and GAPDH), statistically insignificant increases SLC2A1 (GLUT-1) or greatly enhance transcription (VEGFA), but in an HIF-1α-independent manner, as quantified by PCR analysis in cells with shRNA-mediated silencing of HIF-1α. Instead, HIF-1α knockdown facilitated G1/S progression and increased Cyclin D1 protein half-life, via a post-translational pathway. Taken together, these findings link *H. pylori*-induced PI3K-mTOR activation to HIF-1α induced G0/G1 cell cycle arrest by a Cyclin D1-dependent mechanism. Thus, HIF-1α is identified here as a mediator between survival and cell cycle arrest signaling activated by *H. pylori* infection.

## Introduction

*Helicobacter pylori* is a microaerophilic, Gram-negative bacterium that colonizes the human stomach, infecting about 50% population worldwide (Peleteiro et al., 2014). This infection is linked to the development of several gastric pathologies, including chronic atrophic gastritis, gastric, and duodenal ulcer, MALT lymphoma and gastric adenocarcinoma (Atherton, 2006; Correa and Houghton, 2007). *H. pylori* infection contributes to the etiology of these diseases by inducing seemingly contradictory epithelial gastric cell responses, including exacerbated apoptosis and proliferation. In conjunction, these responses lead to disturbances in the normal turnover of gastric epithelium (Jang and Kim, 2000), which favor atrophy, a precursor lesion in the sequence of events leading to intestinal metaplasia, dysplasia, and eventually cancer. Bearing this in mind, it becomes important to understand better the underlying molecular events that initiate such changes.

Consistent with the aforementioned ambiguity in host cell responses, the bacteria activate signaling pathways linked to cell cycle progression and therefore, proliferation, as well as those that result in cell cycle arrest and *H. pylori*-induced apoptosis (Shirin et al., 1999; Ahmed et al., 2000; Suzuki et al., 2009; Tabassam et al., 2009). With respect to the signaling events linked to *H. pylori*-induced cell cycle progression, the PI3K/Akt pathway has received considerable attention. In the context of infection, activation of this pathway is associated with an increase in proliferation via induction of β-catenin/Tcf-Lef-dependent transcription, cell survival, and cell migration, as well as enhanced protein synthesis via mTOR activation (Nagy et al., 2009; Suzuki et al., 2009; Sokolova et al., 2014). However, despite *H. pylori*-induced PI3K activation, the infection promotes opposite effects in gastric cells, such as cell cycle arrest in G1 phase and apoptotic cell death (Shirin et al., 1999; Ahmed et al., 2000) via mechanisms that may involve loss of Survivin (Valenzuela et al., 2010, 2013) amongst others.

On the other hand, *H. pylori* also promotes the induction of Hypoxia Inducible Factor 1α (HIF-1α), the inducible subunit of the heterodimeric transcriptional factor HIF-1. Generally HIF-1 increases in hypoxia where it promotes the expression of genes linked to several cell responses, including increased glycolytic metabolism, angiogenesis, survival, and epithelial-mesenchymal transition, all of which are important for tumor progression (Semenza, 2012). As such, activation of HIF-1α is considered a key step that favors malignant disease progression, also in gastric cancer (Kitajima and Miyazaki, 2013). The canonical signaling pathway controlling HIF-1α expression in normoxia involves the hydroxylation on proline residues (Pro402 and Pro564 in human HIF-1α) by proline hydroxylases and subsequent degradation via the proteasome pathway. In hypoxia, lack of oxygen leads to a decline in proline hydroxylase activity, reduced degradation and as a consequence an increase in HIF-1α protein (Semenza, 2012). However, in addition to hypoxia, HIF-1α can be induced by hypoxia-independent mechanisms involving activation of tyrosine kinase receptors and the downstream PI3K/Akt/mTOR and MEK/ERK pathways, as well as by the production of reactive oxygen species (ROS) (Laughner et al., 2001; Fukuda et al., 2002). Particularly *H. pylori* infection of the gastric epithelium and ROS-mediated stabilization of HIF-1α have been suggested to induce proliferation, inhibit cell death and ultimately favor carcinogenesis in the gastric epithelium (Koshiji et al., 2005; Kitajima and Miyazaki, 2013).

Although HIF-1α is induced by these pathways known to favor tumor development and tumor progression, in more recent years a non-transcriptional function for this protein that contrasts with its canonical role has been described, whereby the protein prevents cell cycle progression by blocking DNA replication and modifying the expression of several proteins involved in cell cycle control, which results in inhibition of the G1/S transition (Goda et al., 2003; Koshiji et al., 2004; Hubbi et al., 2013). Such non-transcriptional HIF-1α activity has been reported in hypoxia, but it remained unknown if this occurs in the context of HIF-1α induction by *H. pylori* infection of gastric cells and what the consequences might be.

Considering that *H. pylori* promotes both HIF-1α induction and PI3K/Akt pathway activation, but also can leads to cell cycle arrest, we hypothesized that HIF-1α may serve as a molecular switch from proliferative signaling towards cell cycle arrest in gastric cells infected with this bacterium. In this study, we evaluated if *H. pylori* promoted PI3K/Akt/mTOR activation, whether this increased HIF-1α protein levels and whether this factor contributed to cell cycle arrest mediated by *H. pylori*. Our results implicate PI3K and mTOR activation in *H. pylori*-triggered HIF-1α induction and G0/G1 cell cycle arrest in infected gastric cells.

## Methods

### Cell lines and culture conditions

The gastric cancer cell lines AGS (ATCC CRL-1793), MKN74 (JCRB0255), and KATO III (ATCC HTB-103) were cultured in RPMI 1640 medium (Gibco) supplemented with 10% Fetal Bovine Serum (Biological Industries) and antibiotics (10,000 U/ml penicillin, 10 μg/ml streptomycin). Cells were incubated at 37°C in a humidified atmosphere with 5% CO_2_.

### *H. pylori* strain and culture conditions

The completely sequenced *H. pylori* 26695 (ATCC 700392) strain was cultured in trypticase soy agar plates supplemented with 5% horse serum (Biological Industries), nutritive supplement Vitox (Oxoid) and selective supplement Dent (Oxoid) for 24 h at 37°C in a humidified atmosphere with 5% CO_2_.

### Transfection of AGS cells

AGS cells were transfected with sh-RNA-Scramble plasmid (Santa Cruz Biotechnology #108065) or sh-RNA-HIF-1α plasmid (Santa Cruz Biotechnology #35561) using the reagent Fugene 6®, following instructions provided by the manufacturer (Promega, Madison, WI). Post-transfection, cells were grown in a selection medium containing 2 μg/ml puromycin for 2 weeks. Then, clones were isolated from batch-transfected cell populations in order to obtain cells with improved silencing of HIF-1α. A clone with effective silencing of HIF-1α (AGS sh-HIF-1α Clone7) and control clone (AGS sh-Scr Clone2) were selected (see Supplementary Figure 5) and used to perform the indicated experiments.

### Infection of gastric cells with *H. pylori*

8 × 10^5^ cells were seeded in 60 mm culture plates and cultivated for 24 h. For infection assays, culture media was replaced by serum supplemented RPMI without antibiotics at least 4 h prior to bacterial exposure. The bacteria were harvested, washed, and resuspended in PBS. OD_560_ = 0.4 was considered equivalent to 3 × 10^8^ bacteria. Cells were infected at the multiplicity of infection (MOI) indicated in each experiment.

### Preparation of protein extracts

To obtain the total protein extracts, cells were lysed with a buffer containing 20 mM HEPES pH 7.4, 12.5 μg/ml leupeptin, 10 μg/ml antipain, 100 μg/ml benzamidine, 1 mM phenylmethylsufonyl fluoride, 1 mM sodium orthovanadate and 10 mM sodium fluoride, 0.1% SDS, 0.05% NP-40 (IGEPAL). Then, lysates were sonicated and centrifuged. The resulting supernatants, referred to as total protein extracts, were stored at −20°C.

Nuclear and the cytoplasmatic fractions were essentially obtained as previously described (Valenzuela et al., 2014). Briefly, cells were lysed in a hypotonic buffer containing 10 mM Hepes-KOH pH 7.9, 2 mM MgCl_2_, 0.1 mM EDTA, 10 mM KCl, 0.5% NP-40 (IGEPAL) and supplemented with 1 mM DTT, as well as protease and phosphatase inhibitors (12.5 μg/ml leupeptin, 10 μg/ml antipain, 100 μg/ml benzamidine, 1 mM phenylmethylsufonyl fluoride, 1 mM sodium orthovanadate and 10 mM sodium fluoride). The lysate was centrifuged at 16,000 × g to obtain the cytoplasmic protein fraction in the supernatant and the nuclear protein fraction in the pellet. The nuclear pellet fraction was solubilized in a hypertonic buffer containing 50 mM Hepes-KOH pH 7.9, 2 mM MgCl_2_, 0.1 mM EDTA, 50 mM KCl, 400 mM NaCl, 10% glycerol, supplemented with 1 mM DTT, as well as the protease and phosphatase inhibitors indicated above. The resulting lysate was centrifuged at 16,000 × g to obtain the nuclear proteins in the supernatant.

### Western blot analysis

Proteins (50–75 μg total per lane) were separated in 10% SDS polyacrylamide gels and transferred to a nitrocellulose membrane, as previously described (Valenzuela et al., 2010). Blots were blocked with 5% milk in 0.1%-Tween20 TBS or 5% BSA in 0.1%-Tween20 TBS for phosphorylated proteins. Then, blots were probed with either mouse anti-HIF-1α monoclonal antibody (BD Bioscience #610958, dilution 1:500), mouse anti-β-actin monoclonal antibody (Sigma #A5316, dilution 1:20,000), rabbit anti-phospho-S473-Akt polyclonal antibody (Cell Signaling Biotechnology #4060, dilution 1:1,000), rabbit anti-Akt polyclonal antibody (Cell Signaling Biotechnology #9272, dilution 1:1,000), mouse anti-Cyclin D1 monoclonal antibody (Santa Cruz Biotechnology #20044, dilution 1:1,000), rabbit anti-phospho-S235/236-S6 polyclonal antibody (Cell Signaling #48585, dilution 1:1,000), mouse anti-S6 monoclonal antibody (Cell Signaling Biotechnology #2317, dilution 1:1,000), mouse anti-LAP2A monoclonal antibody (BD Bioscience #611000, dilution 1:3,000) or rabbit anti-HSP90 polyclonal antibody (Santa Cruz Biotechnology #7947, dilution 1:3,000). Primary antibodies were detected with horseradish peroxidase-conjugated anti-mouse (Kirkergaard and Perry Laboratories #214-1806, dilution 1:5,000) or anti-rabbit (Kirkergaard and Perry Laboratories #214-1516, dilution 1:5,000) secondary antibodies and the ECL system.

Protein levels were essentially quantified as previously described (Valenzuela et al., 2013) by scanning densitometric analysis of Western blots using the software UnScan-it v. 6.1. Values obtained for the respective proteins of interest (HIF1a, Cyclin D1) were standardized to β-actin levels in the same lane. Results shown in graphs are averages from at least three different experiments with their respective standard errors of mean (SEM).

### Luciferase reporter assay

To analyze the HIF-1α transcriptional activity, 1.5 × 10^5^ cells were transfected with 0.25 μg of the respective plasmids: pGL3-HRE (HIF-1α reporter kindly provided by Dr. Kaye Williams, University of Manchester (Brown et al., 2006) and pON (β-galactosidase). Post-transfection, after 16 h, culture media was replaced by supplemented RPMI 1640 without antibiotics for inoculation with bacteria. After infection, cells were lysed in a buffer containing 0.1 M KH_2_PO_4_ (pH 7.9), 0.5% Triton X-100, and 1 mM DTT. The resulting lysate was centrifuged at 1640 × g and supernatants were used to measure either luciferase or β-galactosidase activities. Luciferase activity was detected using a luminometer (Synergy HT Multi-Mode Microplate reader, BioTek Instruments, Winooski, VT) after addition of KTME buffer containing 100 mM Tris HCl, 10 mM MgSO_4_, 2 mM EDTA, 5 mM Na_2_ATP, and 0.1 mM luciferin. β-galactosidase activity was determined by spectrophotometry (410 nm) after addition of substrate 2-nitrophenyl-β-D-galactopyranoside (ONPG). The values reported for luciferase activity were standarized to β-galactosidase activity.

### Analysis of mRNA levels by real time reverse transcription PCR

Total RNA was isolated with the reagent TriZOL® following instructions provided by the manufacturer. RNA samples were spectrophotometrically quantified, digested with DNAse I and characterized by electrophoresis in 2% agarose gels (quality control) and then used as templates to generate cDNA.

The real-time quantitative PCR (qPCR) analysis was performed using the Brilliant II SYBR® Green QPCR Master Mix (Agilent Technologies, La Jolla, CA), cDNA template and the following primers: VEGFA: sense primer 5′-ATCCGGGTTTTATCCCTCTTC-3′ and anti-sense primer 5′-TCTCTCTGGAGCTCTTGCTA-3′; GLUT-1: sense primer 5′- AAGGAAGAGAGTCGGCAGAT-3′ and anti-sense primer 5- TCGAAGATGCTCGTGGAGTA-3'; Bcl-xL: sense primer 5- CACTAACCAGAGACGAGACT-3′ and anti-sense primer 5′- GTCCTGTTCTCTTCCACATC-3′; LDHA: sense primer 5′- ACGTCAGCAAGAGGGAGAA-3′ and anti-sense primer 5′- TCTTCCAAGCCACGTAGGT-3′; GAPDH: sense primer 5′- GCGAGATCCCTCCAAAATCA-3′ and anti-sense primer 5′- ATGGTTCACACCCATGACGA-3′; Cyclin D1: sense primer 5′-GGTGAACAAGCTCAAGTGGA-3′ and anti-sense primer 5′-GAGGGCGGATTGGAAATGAA-3′; RPS13 housekeeping gene: sense primer 5′-CTCTCCTTTCGTTGCCTGAT-3′ and anti-sense primer 5′- TGAAGGAGTAAGGCCCTTCT-3′. The PCR reactions were carried out using the Agilent Mx3000P QPCR System (Agilent Technologies, La Jolla, CA) following suggestions of the manufacturer. The relative gene expression levels were calculated by the 2^−ΔΔCT^ method (Livak and Schmittgen, 2001). VEGFA, GLUT-1, Bcl-xL, LDHA, GAPDH, and Cyclin D1 levels mRNA levels were normalized to RPS13 mRNA (housekeeping gene). All data were expressed relative to values obtained for non-infected cells.

### Cell cycle analysis

After infection, cells were incubated with 200 μg/ml gentamycin at 37°C for 1 h to eliminate adherent bacteria. Then, cells were washed, harvested and fixed in chilled methanol at −20°C for 10 min. Fixed cells were treated with 100 μg/ml RNAase A for 30 min and then stained with 10 μg/ml propidium iodide. Cells were analyzed by flow cytometry (FACS Canto, BD) and the DNA content was quantified as propidium iodide fluorescence intensity histograms using the FACSDiva program version 6.1.3.

### PI3K/Akt/mTOR, protein synthesis, and proteasome inhibitors

The PI3K inhibitor LY294002 (Enzo Life Science #BML-ST420-005) was used at concentrations of 1, 5 and 10 μM; the Akt inhibitor VIII (Calbiochem #124018) was used at 1 μM and the mTOR inhibitor Rapamycin (Sigma #R8781) at 50 nM. The inhibitors were added to culture media 30 min previous to infection. All inhibitors were solubilized in DMSO.

To determine the Cyclin D1 half-life, the protein synthesis inhibitor cycloheximide (CHX) 100 μg/ml (Sigma #C0934) was added to infected cells after 8 h of exposure to *H. pylori* and protein extracts were then prepared at the indicated time points after CHX addition.

To determine the role of the proteasome in Cyclin D1 decrease, the proteasome inhibitor MG132 (20 μM) (Enzo Life Science #BML-PI102-005) was added to infected cells after 8 h of infection, in the absence and presence of protein synthesis inhibitor CHX, for 15 min. Then, protein extracts were prepared for western blot analysis.

### Statistical analysis

Results from at least 3 independent experiments were analyzed by applying 1-way ANOVA analysis and Dunnett post-test to compare multiple conditions or Student's *t*-test to compare two conditions, using the GraphPad Prism software (GraphPad Software, San Diego, CA). *P* < 0.05 was considered significant.

## Results

### *H. pylori* promotes transient HIF-1α induction

**Figure 1:**
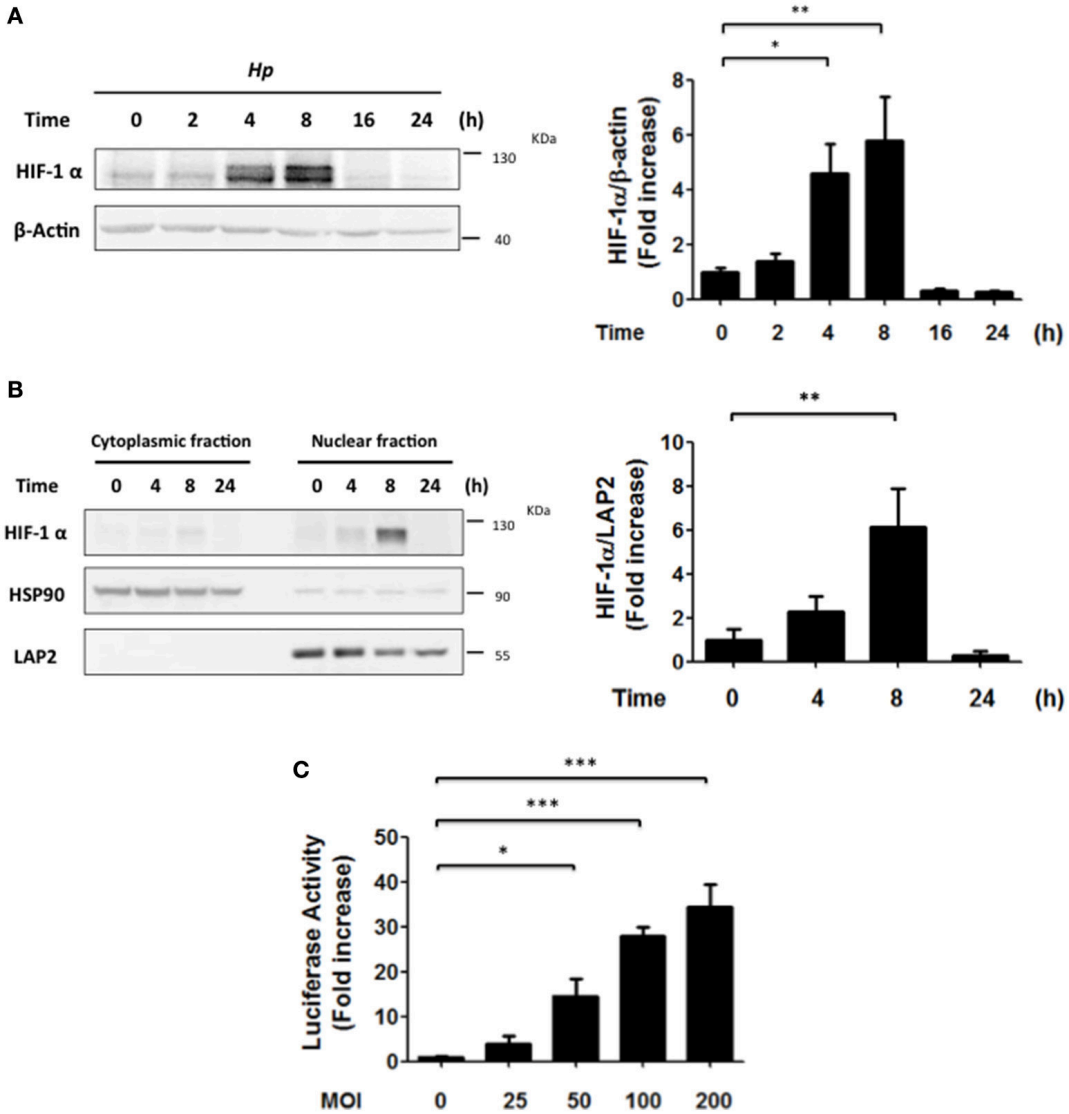
***H. pylori*** **promotes transient HIF-1α induction, nuclear localization and transcriptional activity. (A)** AGS cells were infected with *H. pylori* 26695 at MOI 100 for time periods indicated. The levels of HIF-1α and β-actin were analyzed by western blotting. The relative levels of HIF-1α were determined (mean ± SEM; *n* = 4, ^*^p < 0.05; ^**^p < 0.01). **(B)** AGS cells were infected with *H. pylori* 26695 at MOI 100 for time periods indicated and then cytoplasmic and nuclear fractions were obtained. The levels of HIF-1α, HSP90 (cytoplasmic marker) and LAP2 (nuclear marker) were analyzed by western blotting. The relative nuclear levels of HIF-1α were determined (mean ± SEM, *n* = 4; ^**^p < 0.01). **(C)** AGS cells were co-transfected with the pGL3-HRE-luciferase and pON-β-galactosidase plasmids and then were infected with *H. pylori* at the indicated Multiplicity of Infection (MOI) for 8 h. The cells were lysed and the luciferase and β-galactosidase activities were determined. The luciferase activity was standarized to the β-galactosidase activity (mean ± SEM; *n* = 4; ^*^p < 0.05; ^***^p < 0.001).

Previously, *H. pylori* infection of gastric cells has been linked to HIF-1α induction via ROS-dependent mechanisms, VEGF production and angiogenesis (Park et al., 2003; Bhattacharyya et al., 2010; Kang et al., 2014). However, alternative pathways reportedly also contribute to HIF-1α induction in normoxia, such as activation of PI3K/Akt. Here we evaluated the possibility that PI3K/Akt activation by *H. pylori* might favor HIF-1α induction and sought to identify the cell responses associated with HIF-1 activation. To this end, AGS cells were infected with *H. pylori* 26695 at MOI 100 and the induction of HIF-1α protein levels, as well as its intracellular localization were evaluated by western blotting following infection. HIF-1α protein levels increased until 8 h, but then decreased to essentially basal levels after 16 h of *H. pylori* exposure (Figure 1A). Consistent with this, the nuclear localization of HIF-1α, analyzed by nucleus/cytoplasm fractionation, increased after 8 h, but then decreased at 24 h post-infection (Figure 1B). Similar results were obtained by indirect immunofluorescence analysis of HIF-1α subcellular localization (Supplementary Figure 1). Likewise, *H. pylori*-induced HIF-1α expression also was observed in MKN74 and KATO III gastric cells infected for 8 h, albeit to varying extents (Supplementary Figure 2). We then determined whether HIF-1α induction and translocation to the nucleus were linked to enhanced transcriptional activity, using a luciferase reporter assay. In AGS cells infected with *H. pylori* a MOI-dependent increase in HIF-1 reporter activity was observed after 8 h (Figure 1C).

### PI3K activity is necessary for *H. pylori*-induced Akt activation

**Figure 2:**
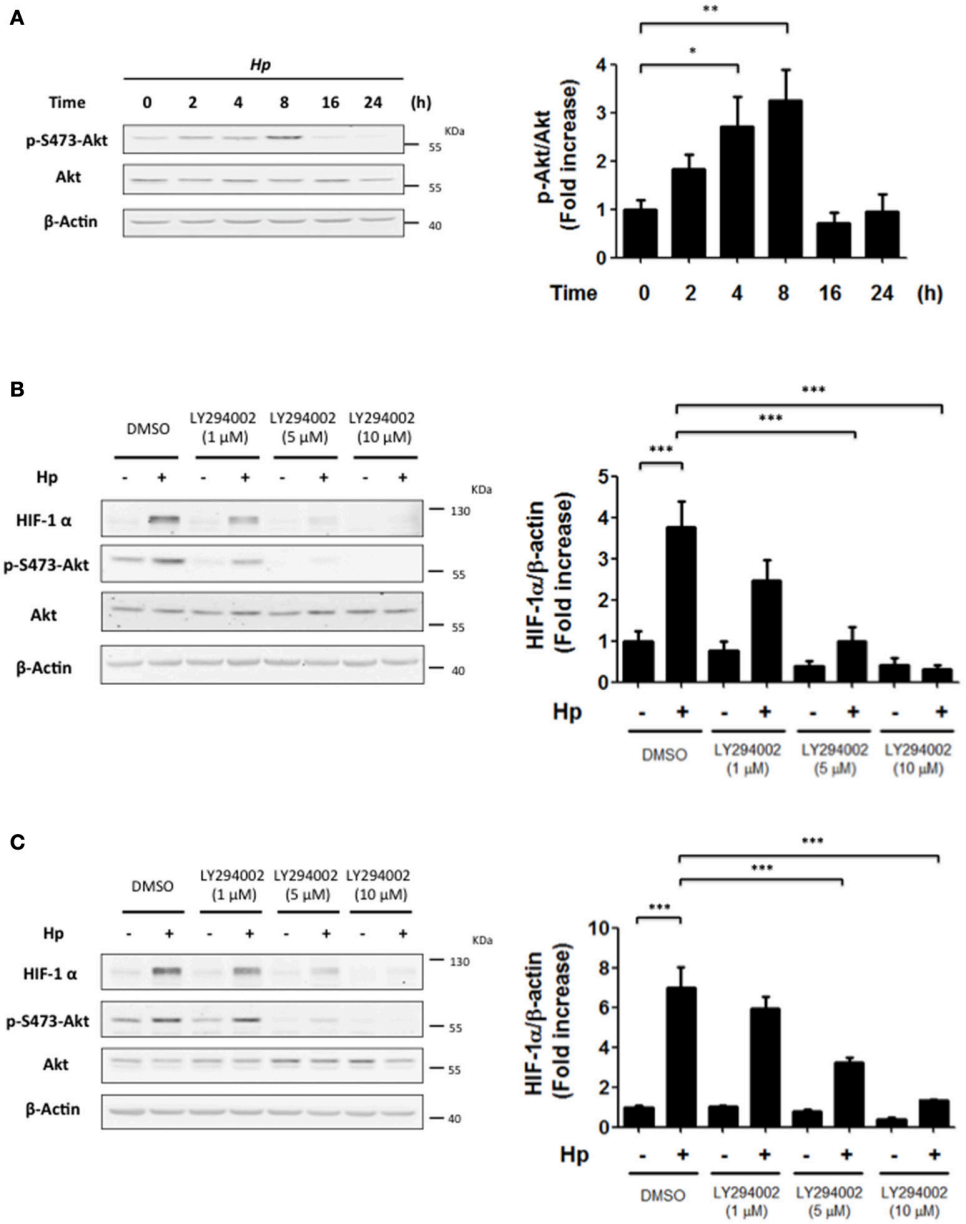
**The inhibition of PI3K precludes the *H. pylori*-promoted HIF-1α induction**. **(A)** AGS cells were infected with *H. pylori* 26695 at MOI 100 for time periods indicated. The levels of p-S473-Akt, total Akt and β-actin were analyzed by western blotting. The relative levels of p-S473-Akt were determined (mean ± SEM; *n* = 4; ^*^p < 0.05; ^**^p < 0.01). **(B,C)** AGS cells were pre-treated with LY294002 (1, 5, and 10 μM) for 30 min and then infected with *H. pylori* 26695 at MOI 100 for 4 h **(B)** or 8 h **(C)**. The levels of HIF-1α, p-S473-Akt, Akt, and β-actin were analyzed by western blotting. The relative levels of HIF-1α/β-actin were determined (mean ± SEM; *n* = 3–4, ^***^p < 0.001).

*H. pylori* infection has been linked to PI3K/Akt activation, which regulates many cellular responses, such as cell cycle progression and proliferation (Nakayama et al., 2009; Suzuki et al., 2009; Tabassam et al., 2009). In normoxia, activation of this pathway downstream of growth factor receptors can induce HIF-1α. Thus, we wondered whether a similar connection might exist following *H. pylori* infection. To that end, the levels of p-Ser473-Akt were evaluated as an indicator of PI3K activity at different time points following infection with *H. pylori*. For AGS cells infected with *H. pylori* 26695 at MOI 100, a substantial increase in p-Ser473-Akt levels was detected after 4 and 8 h of infection, which then decreased 16 and 24 h post-infection (Figure 2A). These changes paralleled those observed for HIF-1α protein levels following *H. pylori* infection (Figure 1A). Thus, PI3K/Akt activation and HIF-1α induction occurred with similar kinetics following *H. pylori* infection.

### PI3K inhibition precludes *H. pylori*-induced HIF-1α expression

To evaluate whether HIF-1α induction was due to PI3K activation, cells were incubated with LY294002, a selective pharmacological inhibitor of PI3K. In AGS cells infected by *H. pylori*, LY294002 (at 5 and 10 μM) reduced significantly HIF-1α induction observed 4 and 8 h post-infection (Figures 2B,C, respectively). Under these conditions, LY294002 prevented PI3K activation by *H. pylori*, since phosphorylation of Akt S473 was almost completely abolished (Figures 2B,C). Alternatively, the adhesion of *H. pylori* to gastric cells was not altered in the presence of the PI3K inhibitor (Supplementary Figures 3A,B), suggesting that the observed results with HIF-1α were not attributable to non-specific effects of the inhibitor on the bacterial adherence to AGS cells. In addition, infection in the presence of the PI3K inhibitor did not affect the translocation of virulence factors to the host cells, since intracellular protein levels of CagA, a virulence factor secreted by the bacteria into the host cell, was not modified by PI3K inhibition (Supplementary Figure 4A). Also, immunoprecipitation of intracellular CagA showed that phosphorylation on tyrosine of CagA, which occurs intracellularly, was not affected by presence of the PI3K inhibitor (Supplementary Figure 4B).

### mTOR inhibition precluded *H. pylori*-promoted HIF-1α induction

**Figure 3:**
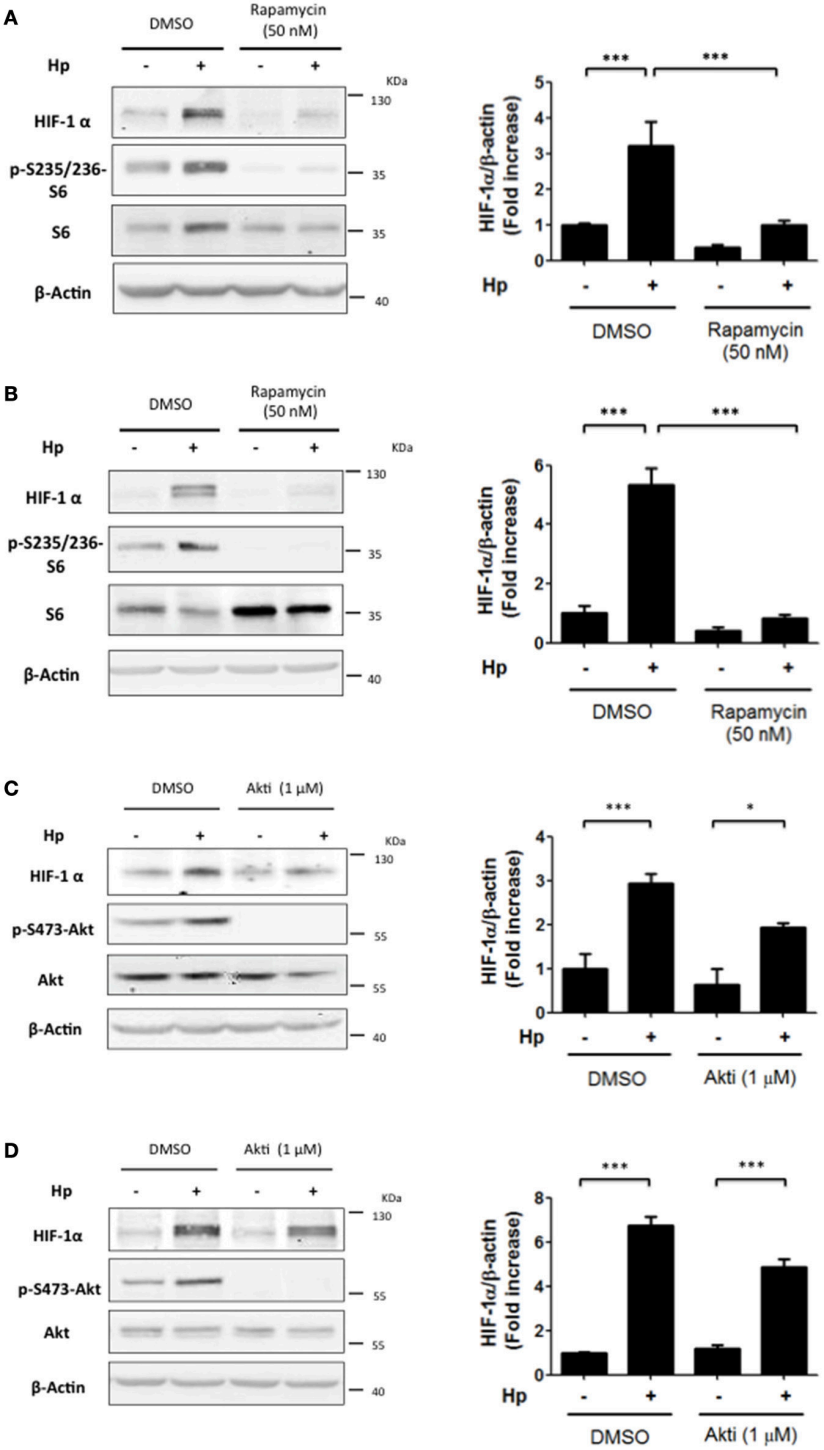
**The inhibition of mTOR precludes the *H. pylori*-promoted HIF-1α induction while Akt inhibition is only partially effective**. **(A,B)** AGS cells were pre-treated with Rapamycin (50 nM) for 30 min and then infected with *H. pylori* 26695 at MOI 100 for 4 h **(A)** or 8 h **(B)**. The levels of HIF-1α, p-S235/236-S6, S6, and β-actin were analyzed by western blotting. The relative levels of HIF-1α were determined (mean ± SEM; *n* = 3–4; ^***^p < 0.001). **(C,D)** AGS cells were pre-treated with Akt inhibitor (1 μM) for 30 min and then infected with *H. pylori* 26695 at MOI 100 for 4 h **(C)** or 8 h **(D)**. The levels of HIF-1α, p-S473-Akt, total Akt, and β-actin were analyzed by western blotting. The relative levels of HIF-1α were determined (mean ± SEM; *n* = 3.4, ^*^p < 0.05; ^***^p < 0.001).

In fibroblasts, as well as breast and colon cancer cells, stimuli leading to PI3K activation increase HIF-1α protein levels through the downstream activation of Akt and mTOR (Laughner et al., 2001; Fukuda et al., 2002). To determine which elements downstream of PI3K were required for *H. pylori*-mediated HIF-1α induction, cells were incubated with the mTOR inhibitor Rapamycin (50 nM) during infection by 4 h or 8 h and mTOR inhibition was assessed by determining the levels of S6 phosphorylation on S235/236 (p-S235/236-S6) due to S6K, a direct target of mTOR (Semenza, 2012). Indeed, Rapamycin for either 4 h or 8 h effectively inhibited mTOR activity and precluded the *H. pylori*-induced HIF-1α protein levels in AGS cells (Figures 3A,B, respectively). Again, as a control, Rapamycin (50 nM) was shown neither to affect *H. pylori* adhesion to AGS cells (Supplementary Figure 3C,D), or the phosphorylation of immunoprecipitated CagA (Supplementary Figure 4C). Interestingly, the Akt inhibitor (1 μM) was not significantly effective in reducing HIF-1α induction following 4 or 8 h *H. pylori* infection (Figures 3C,D, respectively), suggesting that mTOR activation downstream of PI3K is the major pathway responsible for HIF-1α induction following *H. pylori* infection.

### *H. pylori* infection does not change HIF-1α target gene expression

**Figure 4:**
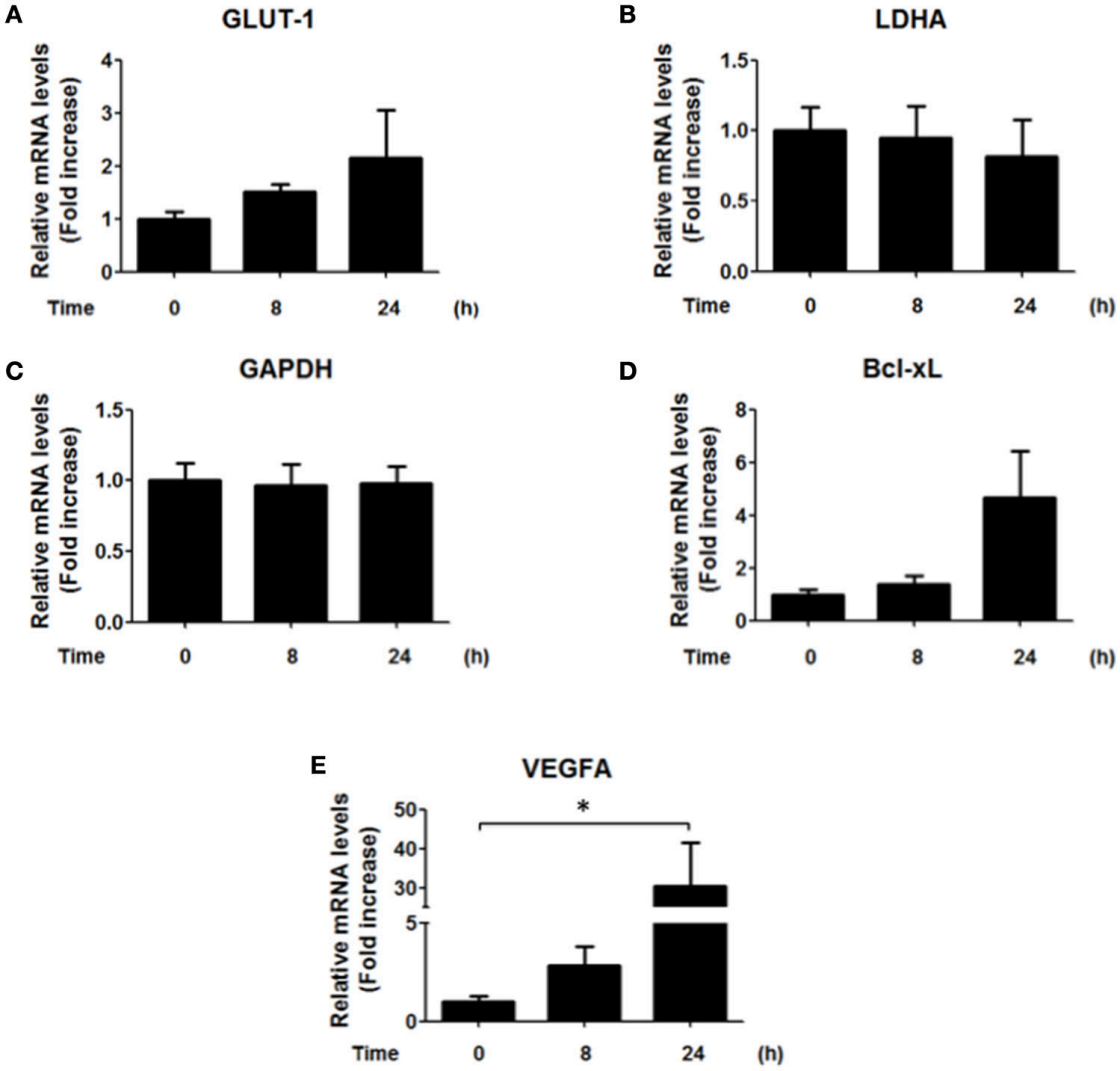
**Effect of *H. pylori* infection on HIF-1α target gene expression**. AGS cells were infected with *H. pylori* 26695 at MOI 100 for 8 h and 24 h. mRNA levels of GLUT-1 **(A)**, LDHA **(B)**, GAPDH **(C)**, Bcl-xL **(D)**, and VEGFA **(E)** were evaluated by RT-qPCR. The levels of each mRNA were normalized to RNA of the RPS13 housekeeping gene. All data were expressed relative to values obtained for time 0 h of infection (mean ±SEM; *n* = 3–4; ^*^p < 0.05).

Since our results showed an increase in the nuclear localization of HIF-1α and an increase in the reporter activity after 8 h of infection by *H. pylori* (Figures 1B,C), we next evaluated whether some of the known target genes of HIF-1α were induced by *H. pylori* infection. Unexpectedly, *H. pylori* promoted only few and rather modest changes in the mRNA levels of known downstream target genes of HIF-1α. Specifically, GLUT-1, LDHA, and GAPDH did not change, while for Bcl-xL a trend but not a significant increase was observed (Figure 4). This suggested to us that HIF-1α induction following *H. pylori* infection may be more closely linked to non-canonical functions.

**Figure 5:**
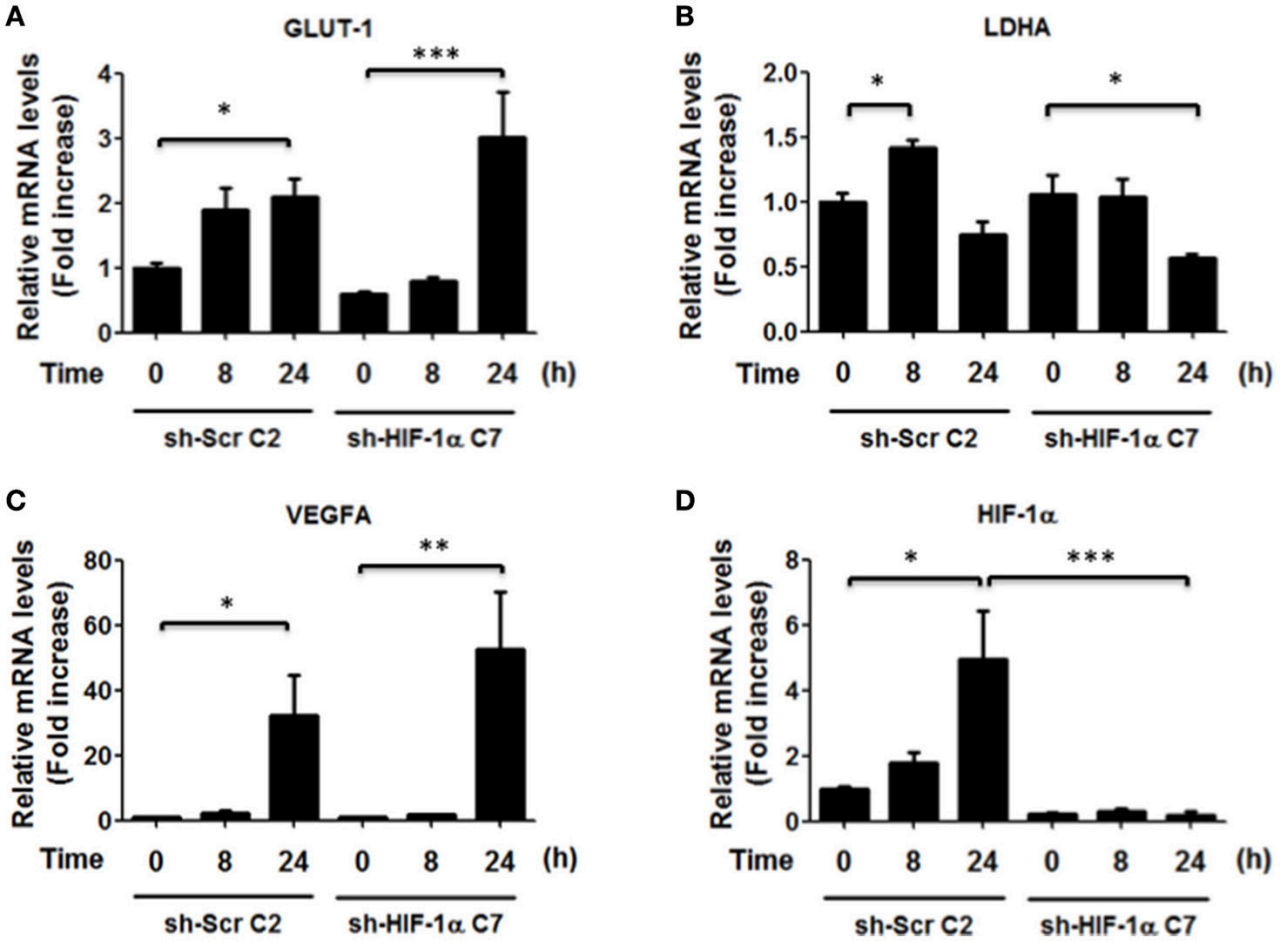
**Effect of HIF-1α silencing on gene induction in response to *H. pylori* infection**. AGS sh-Scr C2 and sh-HIF-1α C7 cells were infected with *H. pylori* 26695 at MOI 100 for 8 and 24 h. mRNA levels of GLUT-1 **(A)**, LDHA **(B)**, VEGFA **(C)**, and HIF-1α **(D)** were evaluated by RT-qPCR. The levels of each mRNA were normalized to RNA of the RPS13 housekeeping gene. All data were expressed relative to values obtained for time 0 h of infection in sh-Scr cells (mean ± SEM; *n* = 3–4, ^*^p < 0.05; ^**^p < 0.01; ^***^p < 0.001).

Thus, to determine whether HIF-1α was required to increase transcription of the indicated genes, we also isolated clonal populations of AGS cells in which HIF-1α had been silenced. AGS cells were transfected with sh-RNA that specifically silenced HIF-1α expression or with a sh-RNA Scramble as a control and then infected with *H. pylori*. To evaluate the efficiency of the HIF-1α silencing, stably transfected cells were treated with the chemical inducer of HIF-1α, Deferroxamine (DFO) (200 μg/ml), and the levels of HIF-1α were determined by western blotting (Supplementary Figure 5). The stably transfected clones AGS sh-HIF-1α C7 and AGS sh-Scr C2 were chosen for infection by *H. pylori* 26695 at MOI 100 for 24 h and induction of mRNA of several HIF-1α target genes were evaluated. In these AGS sh-Scr C2 (clonal control) cells, some differences with respect to the wild-type cells were noted. For instance, we observed modest changes in the mRNA levels of GLUT-1 after 24 h of *H. pylori* infection. Importantly, however, similar changes were also detected in sh-HIF-1α C7 cells lacking HIF-1α, indicating that they were HIF-1α-independent. For LDHA, a minor increase was observed after 8 h of bacterial infection in cells expressing HIF-1α. However, overall a trend toward decreased LDHA was detectable after 24 h both in the cells expressing or lacking HIF-1α (Figure 5). Moreover, for VEGFA substantially elevated expression was noticeable after 24 h, but again these changes were seen in cells expressing or not HIF-1α (Figure 5). Taken together, these results suggested that the increase in nuclear HIF-1α protein levels and reporter activity (Figures 1B,C, respectively) was likely to be associated with additional functions beyond the transcriptional activation of common target genes.

### *H. pylori* induces G0/G1 cell cycle arrest in gastric cells

**Figure 6:**
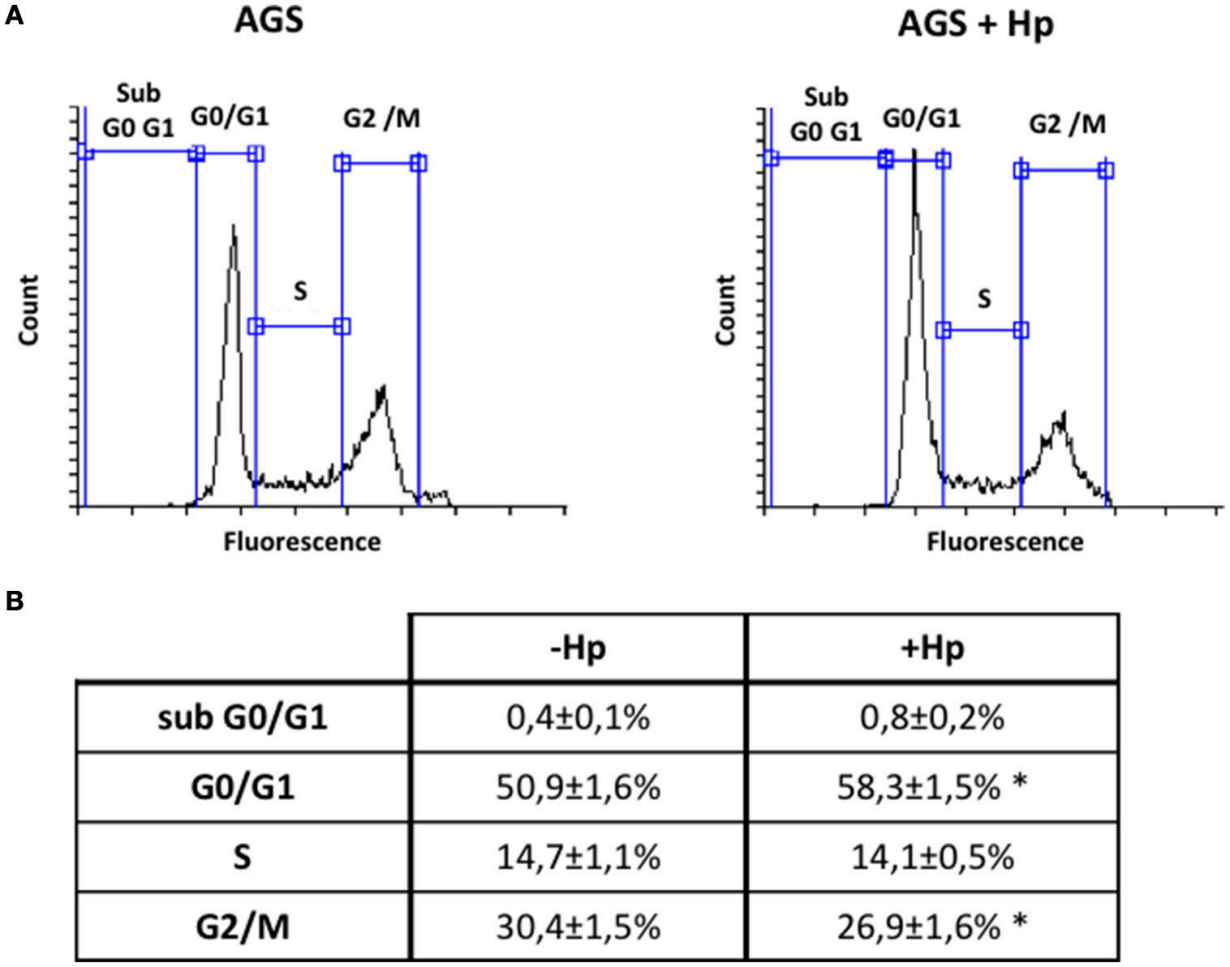
***H. pylori*** **infection increases cells in G0/G1 phase and decreases cells in G2/M phase of cell cycle**. AGS cells were infected with *H. pylori* 26695 at MOI 100 for 24 h and cell cycle distribution was determined by flow cytometry. **(A)** Representative profiles of the cell cycle distribution are shown for the indicated conditions. **(B)** Percentage of cells in each phase of the cell cycle phase are summarized (mean ± SEM, *n* = 4; ^*^p < 0.05).

Although *H. pylori* promotes activation of signaling pathways linked to cell cycle progression and proliferation (Suzuki et al., 2009), the bacteria is also known to induce G1 cell cycle arrest (Shirin et al., 1999; Ahmed et al., 2000). To evaluate in our hands how *H. pylori* modulates the cell cycle, AGS cells were infected with the bacteria at MOI 100 for 24 h. This time point was chosen post-infection considering that it represented roughly the amount of time necessary to complete one cycle of duplication. Then, cell cycle distribution was analyzed by flow cytometry. As is shown (Figure 6), infection increased the number of cells in G0/G1 and decreased the number of cells in G2/M compared to non-treated cells. Alternatively, the number of cells in S phase was not significantly altered by infection.

### HIF-1α silencing prevents G0/G1 cell cycle arrest promoted by *H. pylori* in gastric cells

**Figure 7:**
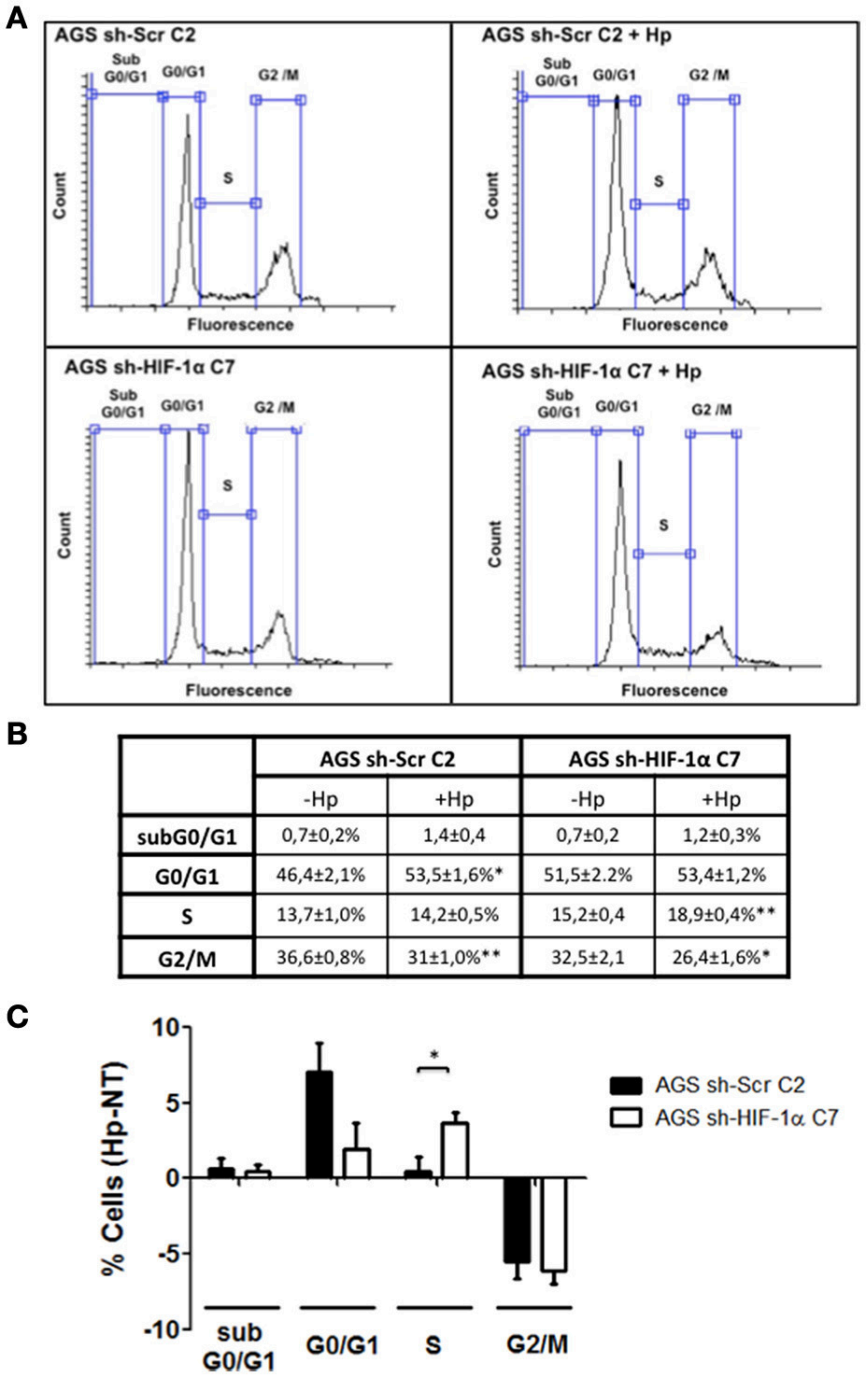
**The lack of HIF-1α promotes the transition from G1 to S phase of the cell cycle in gastric cells infected with *H. pylori***. Stably transfected AGS sh-Scr C2 and AGS sh-HIF-1α C7 cells were infected with *H. pylori* 26695 at MOI 100 for 24 h and cell cycle distribution was determined by flow cytometry. **(A)** Representative cell cycle distribution profiles for the indicated conditions. **(B)** Percentage of cells in each phase of the cell cycle are summarized (mean ± SEM, *n* = 4; ^*^p < 0.05; ^**^p < 0.01) **(C)** Changes following infection in the percentage of cells in each phase of the cell cycle are summarized graphically (mean ± SEM, *n* = 4; ^*^p < 0.05).

Our observations shown in Figure 5 indicated that HIF-1α induction by *H. pylori* may be triggering an alternative response, not necessarily linked to an increase in the expression of specific genes. Indeed, in more recent years, a non-canonical role for HIF-1α, independent of its transcriptional activity, has emerged, involving the inhibition of DNA replication and promotion of G1 cell cycle arrest (Huang, 2013). To test whether *H. pylori*- induced HIF-1α is able to induce cell cycle arrest, the stably transfected clones AGS sh-HIF-1α C7 and AGS sh-Scr C2 were infected by *H. pylori* 26695 at MOI 100 for 24 h and cell cycle distribution was determined by flow cytometry. We observed that infection with *H. pylori* increased the percentage of cells in the G0/G1 phase in sh-Scr C2 AGS cells (Figures 7A–C), while the percentage of cells in S phase increased in sh-HIF-1α C7 AGS cells (Figures 7–C). Moreover, infection decreased the percentage of both control cells and HIF-1α knockdown cells in G2/M phase to the same extent (Figures 7–C). Taken together, these data suggest that *H. pylori*-induced HIF-1α prevents G1/S cell cycle progression, but does not affect transition to the G2/M phase.

### HIF-1α knockdown reduces *H. pylori*-induced loss of cyclin D1 protein

**Figure 8:**
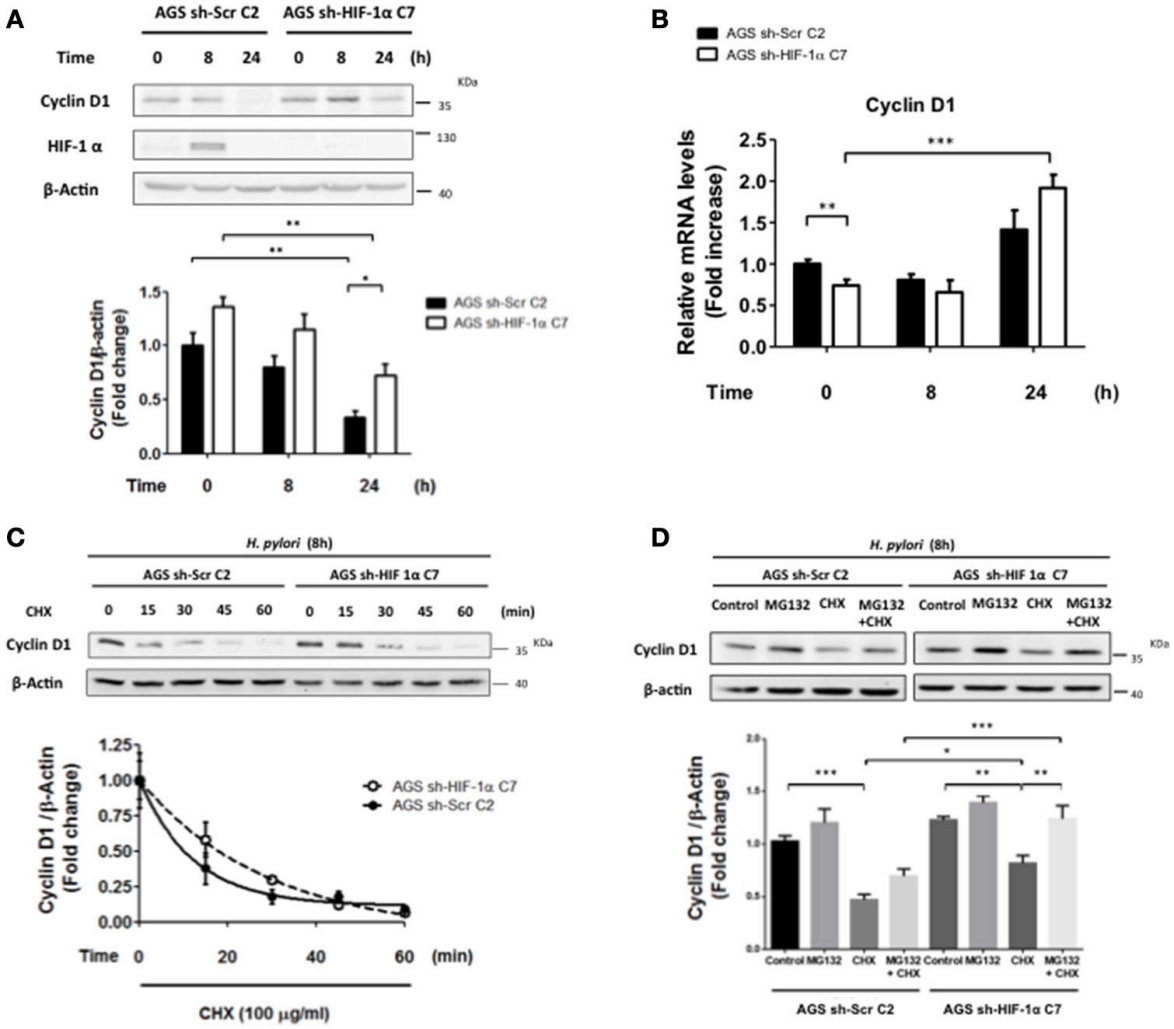
**The lack of HIF-1α partially attenuates *H. pylori*-enhanced Cyclin D1 decline by a post-translational mechanism**. **(A)** Stably transfected AGS sh-Scr C2 and AGS sh- HIF-1α C7 cells were infected with *H. pylori* 26695 at MOI 100 for 8 and 24 h. The levels of Cyclin D1, HIF-1α, and β-actin were analyzed by western blotting. The relative levels of Cyclin D1 were determined (mean ±SEM, *n* = 4; ^*^p < 0.05; ^**^p < 0.01). **(B)** Stably transfected AGS sh-Scr C2 and AGS sh- HIF-1α C7 cells were infected with *H. pylori* 26695 at MOI 100 for 8 and 24 h. mRNA levels of Cyclin D1 were evaluated by RT-qPCR. The levels of Cyclin D1 mRNA were normalized to RNA levels of RPS13, employed here as a housekeeping gene. Data were expressed relative to values obtained for time 0 h of infection (mean ± SEM, *n* = 4; ^**^p < 0.01; ^***^p < 0.001). **(C)** Stably transfected AGS sh-Scr C2 and AGS sh-HIF-1α C7 cells were infected with *H. pylori* 26695 at MOI 100 for 8 h and then treated with cycloheximide (100 μg/ml) for the time points indicated. The levels of Cyclin D1 and β-actin were analyzed by western blotting. The relative levels of Cyclin D1 were determined (mean ± SEM, *n* = 3). **(D)** Stably transfected AGS sh-Scr C2 and AGS sh-HIF-1α C7 cells were infected with *H. pylori* 26695 at MOI 100 for 8 h and then cycloheximide (CHX) (100 μg/ml) and/or MG132 (20 μM) were added. Cells extracts were collected after 15 min of these treatments. Cyclin D1 and β-actin levels were evaluated by western blotting. The relative levels of Cyclin D1 were determined (mean ± SEM, *n* = 4; ^*^p < 0.05; ^**^p < 0.01; ^***^p < 0.001).

Cyclin D1 is required for progression of cells through the G1 phase of the cell cycle, and decreased expression has been linked to an arrest in this phase (Malumbres, 2011). To evaluate whether HIF-1α modulated Cyclin D1 levels and promoted cell cycle arrest in G0/G1 phase, sh-HIF-1α C7 and sh-Scr C2 AGS cells were infected with *H. pylori* for 8 and 24 h and Cyclin D1 levels were measured. We observed that Cyclin D1 protein levels decreased in both cell lines after 24 h of infection; however, for HIF-1α knockdown cells, Cyclin D1 levels were higher compared to control cells at this time point of infection (Figure 8A), suggesting that HIF-1α contributes to the decrease in Cyclin D1 triggered by *H. pylori* infection and cell cycle arrest. To evaluate whether the decrease in Cyclin D1 protein levels mediated by *H. pylori*-induced HIF-1α was attributable to a transcriptional mechanism, the Cyclin D1 mRNA in sh-HIF-1α C7 and sh-Scr C2 AGS cells infected with *H. pylori* were determined. Our results revealed that infection did not reduce the transcription of Cyclin D1 in either of the stably transfected cell lines. If anything, a tendency toward increased expression was observed 24 h post-infection by *H. pylori*, and this trend was similar for cells expressing or not HIF-1α (Figure 8B). To analyze whether *H. pylori*-induced HIF-1α affected Cyclin D1 at the post-translational level, sh-HIF-1α C7 and sh-Scr C2 AGS cells were infected by *H. pylori* for 8 h, treated with cycloheximide (CHX) to block the protein synthesis and then the kinetics of Cyclin D1 protein loss were determined. As shown in Figure 8C, Cyclin D1 protein levels declined more rapidly for the sh-Scr C2 than for sh-HIF-1α C7 AGS cells. Protein half-life was found to be approximately 9 min (t_1/2_ = 9 min) in sh-Scr C2 AGS cells, compared to the 20 min (t_1/2_ = 20 min) for sh-HIF-1α C7 cells. These results suggest that *H. pylori*-induced HIF-1α promotes the decline in Cyclin D1 by a post-translational mechanism.

Finally, to determine whether the proteasome pathway is involved in this mechanism, AGS cells infected with *H. pylori* for 8 h were treated with CHX together with MG132, a specific proteasome inhibitor. Our results showed that infection in the presence of MG132 and CHX partially restored Cyclin D1 levels compared to the treatment with CHX alone (Figure 8D), suggesting that Cyclin D1 decrease mediated by *H. pylori* infection is attributable in part to degradation via the proteasome. This recovery is apparently more effective in AGS sh-HIF-1α C7 cells; however, the degree of recovery (expressed in percent) in Cyclin D1 in the presence of MG132 is similar for both AGS sh-Scr C2 and sh-HIF-1α C7 cells (Figure 8D). This can be taken to suggest that the presence of HIF1α does not alter proteasome-mediated turnover, but rather some other post-translational process.

## Discussion

The infection with *H. pylori* has been associated with seemingly contradictory responses in human gastric samples and *in vitro* studies. While infection with the bacteria reportedly activates proliferative and anti-apoptotic signaling, also apoptosis and cell cycle arrest have been observed (Shirin et al., 1999; Ahmed et al., 2000; Nagy et al., 2009; Suzuki et al., 2009; Tabassam et al., 2009; Valenzuela et al., 2010, 2013). In the present study, HIF-1α was identified as a molecular linker between proliferative and anti-proliferative effects, since activation of PI3K/mTOR, a well-established proliferative and survival pathway, following *H. pylori* infection, lead to increased HIF-1α expression, which in turn promoted G0/G1 cell cycle arrest in gastric cells.

*H. pylori*-mediated HIF-1α induction is generally attributed to oxidative stress produced by the bacteria in gastric cells (Park et al., 2003; Bhattacharyya et al., 2010; Kang et al., 2014). Here, however, we showed that HIF-1α induction was due to *H. pylori*-induced PI3K/mTOR activation. Both mechanisms are not exclusive, but rather may be complementary, considering that oxidative stress activates many kinases, including PI3K (Schieber and Chandel, 2014). Indeed, we have previously reported on the possible role of reactive oxygen species produced due to liberation of gamma glutamyl transpeptidase (GGT) by the bacteria in down-regulation of Survivin following infection of gastric cells (Valenzuela et al., 2013). In those studies, we generated an *H. pylori* strain lacking GGT activity (Δ*ggt*) and observed that such bacteria failed to induce the formation of reactive oxygen species upon infection of recipient gastric cells. Interestingly, however, Δ*ggt H. pylori* were still able to induce HIF-1α upon infection of cells (data not shown). Thus, additional experiments will be necessary to determine how PI3K is activated during *H. pylori* infection.

Our studies showed that *H. pylori*-induced PI3K/Akt activation is transient, occurs relatively early on following infection and follows kinetics similar to those observed for HIF-1α induction. As anticipated, inhibition of PI3K completely abolished HIF-1α induction; however, rather unexpectedly, experiments using the Akt inhibitor (Akti) were not effective, while the mTOR inhibitor Rapamycin emerged as being more relevant in this respect. Indeed, in a melanoma cell model PI3K inhibition was recently shown to prevent the activation of mTOR and cellular proliferation, but Akt inhibition did not lead to the same effect (Silva et al., 2014). These reported findings together with our own observations support the notion that mTOR activation may occur downstream of PI3K in an Akt-independent manner.

*In vitro* studies have demonstrated that both cell cycle progression and cell cycle arrest are favored following *H. pylori* infection. However, there are notable temporal differences: while infection tends to favor cell cycle progression early on, G1 cell cycle arrest and apoptosis are often the consequence at later stages of infection (Peek et al., 1999; Shirin et al., 1999). Consistent with this interpretation, our studies showed that PI3K/Akt activation occurs relatively rapidly post-infection, but then cell cycle arrest was observed later on. Interestingly, however, HIF-1α also was induced at early times of infection, but participates as shown here in *H. pylori*-mediated inhibition of the G1/S transition, indicating that infection-induced G0/G1 cell cycle arrest is not a late event, but rather a phenomenon that is initiated early on post-infection. The importance of our findings resides in showing that HIF-1α, a protein normally associated with survival responses, particularly downstream of PI3K activation, participates in the transition to cell cycle arrest.

Beyond the HIF-1α-mediated arrest in G1/G0 phase following infection, the percentage of cells in G2/M phase decreased, suggesting that additional effects of *H. pylori* on the cell cycle occur in a manner independent of HIF-1α. Indeed, alterations in the G2/M phase may be related to the decrease in expression of the anti-apoptotic protein Survivin following *H. pylori* infection, as we previously reported (Valenzuela et al., 2010, 2013). However, Survivin reduction is not likely linked to HIF-1α in our experimental conditions, because HIF-1α would be expected to promote transcription of BIRC5 (Survivin) and other genes (Peng et al., 2006; Semenza, 2012). None-the-less, despite increased reporter activity (Figure 1C), we only observed relatively modest increases in target gene expression (see Figure 4) that were largely independent of HIF-1α induction (Figure 5). Likewise, loss of Survivin expression following *H. pylori* infection was similar after 24 h in AGS cells expressing or not HIF-1α (data not shown). Taken together, these observations suggest that *H. pylori*-induced Survivin decline may not only promote apoptotic cell death, but also decrease the number of cells in the G2/M phase in a manner independent of HIF-1α.

As indicated above, we previously showed that *H. pylori* infection induces apoptosis in infected cells after 24 h (Valenzuela et al., 2010, 2013) and we corroborated here that viability decreased by roughly 40–50% under these conditions (Supplementary Figure 6). Thus, a concern in our studies could have been the fact that HIF-1α expression peaked 8 h after *H. pylori* infection and disappeared subsequently. Specifically, the apparently non-canonical role documented here for HIF-1α after 24 h could have been attributed to the fact that induction of HIF-1α only occured in a fraction of the infected AGS cells and that those expressing HIF-1α selectively underwent apoptosis leaving only cells in which HIF-1α expression had never been induced. This can be considered a highly unlikely possibility for a number of reasons. First, the multiplicity of infection (MOI 100) employed was such that all cells should have been infected by the bacteria. Second, as far as we were able to determine, HIF1α was induced to a similar extent in most cells after 8 h. Indeed, using indirect immunofluorescence analysis we found that about 80% of the nuclei were HIF1α positive (Supplementary Figure 1C), but viability only decreased by 40–50% after 24 h (Supplementary Figure 6). Third, loss in viability was the same for sh-Scr C2 and sh-HIF-1α C7 AGS cells (Supplementary Figure 6). Taken together, these findings make it highly unlikely that we were selectively eliminating HIF1α expressing cells following infection with *H. pylori* and therefore, that the effects observed after 24 h were not linked to induction of HIF-1α upon *H. pylori* infection.

*H. pylori*-induced HIF-1α expression is associated with increased expression of Mcl-1, VEGF and Lon protease, via a transcriptional mechanism (Bhattacharyya et al., 2010; Kang et al., 2014; Luo et al., 2016). However, in our hands, we found little evidence for enhanced transcription of typical target genes due to HIF-1α (see Figures 4, 5), despite the notable increase in HIF-1α protein levels and reporter activity (Figure 1C). Thus, our results favor the notion that the G0/G1 cell cycle arrest due to HIF-1α induction is part of a non-transcriptional mechanism, as has been suggested in other experimental contexts (Goda et al., 2003; Koshiji et al., 2004; Hubbi et al., 2013). Although HIF-1α reportedly is pro-carcinogenic, it was found here to promote cell cycle arrest, which may potentially be relevant in early steps of gastric cancer development (Xia and Talley, 2001). Together, these observations suggest that HIF-1α reduces the proliferation that would be necessary to offset apoptosis seen also under our experimental conditions (see Supplementary Figure 6). These observations may be relevant to those known to occur in the gastric epithelium following *H. pylori* infection and the development of pre-neoplastic lesions in atrophic gastritis (Xia and Talley, 2001; Correa and Houghton, 2007).

Previous studies have suggested that *H. pylori* infection induces the expression of Cyclin D1 via activation of the mitogen-activated protein kinase (MAPK) pathway (Hirata et al., 2001). Consistent with these findings, we did see a trend toward increased Cyclin D1 mRNA levels that became statistically significant in those cells where HIF-1α had been knocked down using specific shRNA constructs after 24 h of infection (Figure 8B). Despite this increase, Cyclin D1 protein levels decreased, likely due to enhanced protein degradation as is suggested by the notable decline in Cyclin D1 protein half-life in sh-Scr C2 AGS cells compared to that of the sh-HIF-1α C7 cells lacking HIF-1α (see Figure 8). Cyclin D1 activates Cdk4 and Cdk6, both of which are important in cell cycle progression through the G1 phase, and reduced Cyclin D1 expression impedes the G1/S transition (Malumbres, 2011). Thus, the observed G1/G0 cell cycle arrest attributable here to the *H. pylori*-induced HIF-1α increase is likely to reflect the more rapid decrease in Cyclin D1 protein (reduced protein half-life) in cells expressing HIF-1α.

Cyclin D1 protein levels reportedly decrease following insult by genotoxic agents (Alao, 2007) and *H. pylori* has been shown to induce DNA damage (Toller et al., 2011). Moreover, HIF-1α has been suggested to favor genomic instability by inhibiting proteins necessary for DNA repair in a manner independent of its transcriptional activity (Koshiji et al., 2005; Yoo et al., 2011). These mechanisms would provide an explanation for previous studies showing that HIF-1α induction following *H. pylori* infection results in DNA damage, which in turn would be expected to reduce Cyclin D1 levels, as observed in our experiments. However, in our hands we were unable to detect any significant differences in DNA damage, as assessed by the comet assay, following *H. pylori* infection of cells expressing or not HIF-1α (see Supplementary Figure 7).

HIF-1α reportedly prevents Cyclin D1 expression by binding to the promoter region of the gene (Wen et al., 2010). Consistent with this possibility, we did observe that HIF-1α silencing augmented Cyclin D1 mRNA after infection (Figure 8B). However, Cyclin D1 mRNA levels did not decrease in infected cells expressing HIF-1α, allowing us to rule out the possibility that HIF-1α reduces Cyclin D1 expression by this transcriptional mechanism in our model. Instead, Cyclin D1 half-life in the presence of bacteria was reduced in sh-Scr C2 AGS cells compared to that of the sh-HIF-1α C7 cells lacking HIF-1α. Thus, HIF-1α promotes Cyclin D1 decrease by a post-translational mechanism. Unfortunately, although the proteasome pathway plays a prevalent role in Cyclin D1 turnover (Alao, 2007), and also was found to participate in our experimental system, results obtained with the proteasome inhibitor MG132 indicated that differences in Cyclin D1 protein half-life in the presence or absence of HIF-1α were not likely attributable to modulation of the proteasome pathway.

**Figure 9:**
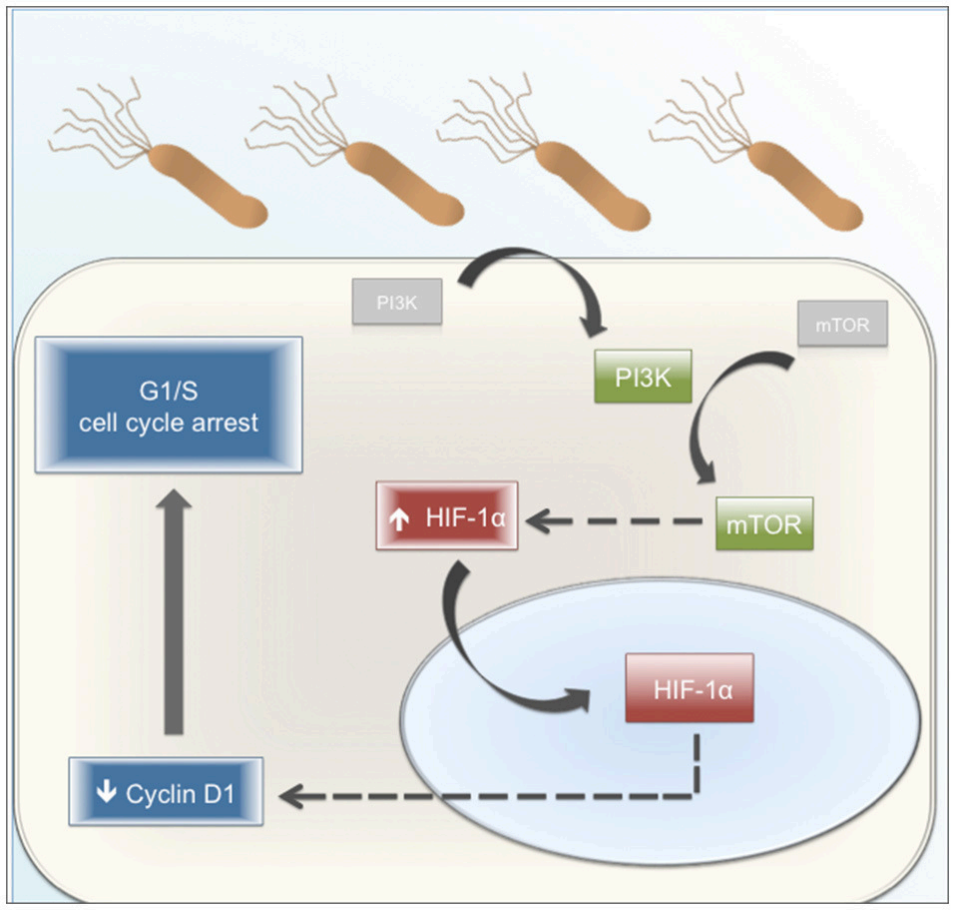
**Proposed working model**. *H. pylori* infection promotes the activation of PI3K and subsequently, of mTOR. The activation of this pathway increases the levels of HIF-1α, a protein mainly present in the nucleus. *H. pylori*-induced HIF-1α decreases the Cyclin D1 levels by a post-translational mechanism. Cyclin D1 is a protein necessary for G1 phase progression. Therefore, its reduction affects the normal progression of the cell cycle, promoting an arrest in G1 phase. Gray boxes: Inactive protein kinases. Green boxes: Active protein kinases associated with cell cycle progression. Blue boxes: effects linked to cell cycle arrest. Red boxes: HIF-1α as a switch between cell cycle progression and cell cycle arrest. Dashed arrows: steps ocurring by mechanism(s) that were not defined in this study.

In conclusion, the results obtained in this study uncover a novel role for HIF-1α in response to *H. pylori* infection of gastric cells, serving to connect an initial early potentially survival response, downstream of PI3K/mTOR activation, to G1/G0 cell cycle arrest later on following infection. The sequence of events is summarized in a tentative working model (Figure 9). Infection with *H. pylori* initially triggers PI3K activation, by as yet undefined mechanisms. Downstream activation of mTOR is depicted as stabilizing HIF-1α protein, which translocates to the nucleus, where instead of promoting transcription of target genes it participates in destabilizing Cyclin D1 protein and thereby favoring G1/S cell cycle arrest. Thus, HIF-1α emerges as an unsuspected linker between cell cycle progression/proliferation and cell cycle arrest/apoptosis, two seemingly contradictory effects observed in gastric cells following infection with *H. pylori*. Further studies are required to understand the *H. pylori*–induced mechanisms that control this switch in HIF-1α function from a transcription factor that generally is thought to promote cell survival to a factor that favors cell cycle arrest.

## Author contributions

JC, DB, and AQ designed research. JC, MV, PC, and JB performed research, acquired and analyzed data. JC, HT, MV, DB, and AQ interpreted data. JC, MV, DB, and AQ wrote the manuscript. All authors provided critical revision to the manuscript and have approved the final version. All authors agree to take responsibility for accuracy and integrity of the research.

### Conflict of interest statement

The authors declare that the research was conducted in the absence of any commercial or financial relationships that could be construed as a potential conflict of interest.
