# Supplementary figures and images for: Helicobacter pylori Induced Phosphatidylinositol-3-OH Kinase/mTOR Activation Increases Hypoxia Inducible Factor-1α to Promote Loss of Cyclin D1 and G0/G1 Cell Cycle Arrest in Human Gastric Cells
Source: Front Cell Infect Microbiol. 2017 Mar 28;7:92. doi: 10.3389/fcimb.2017.00092 (PMC5368181; doi:10.3389/fcimb.2017.00092)

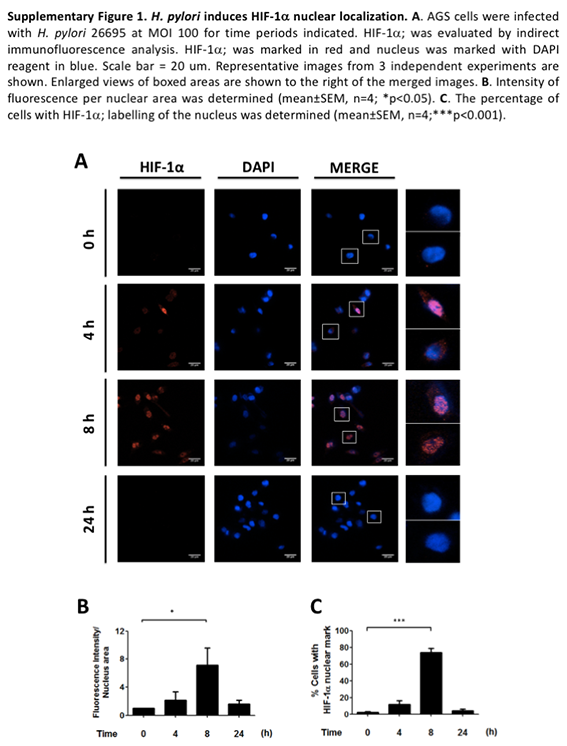

Supplement: Supplementary file 1 [file Image1.TIFF]

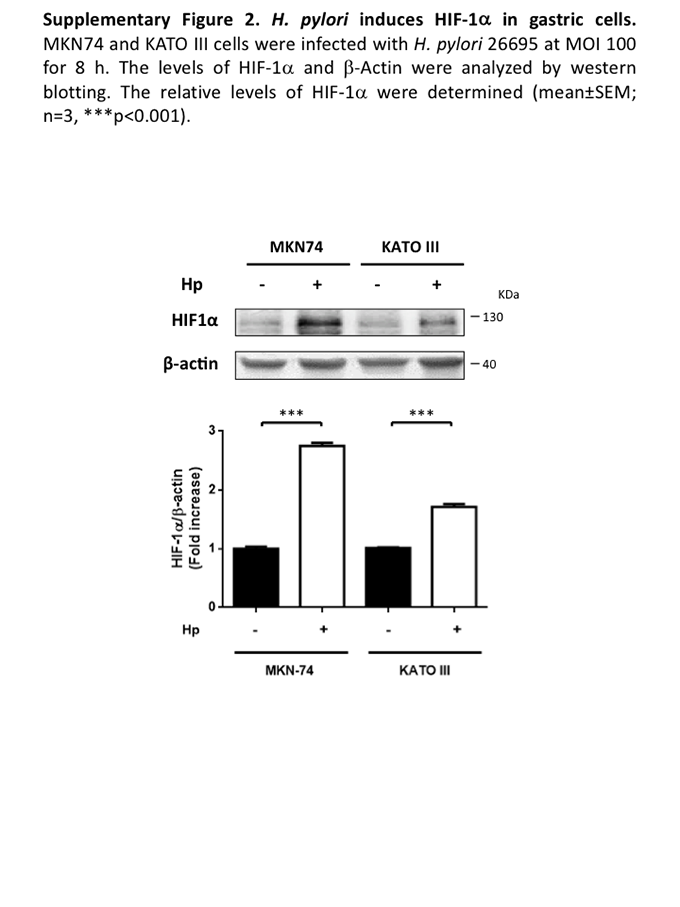

Supplement: Supplementary file 2 [file Image2.TIFF]

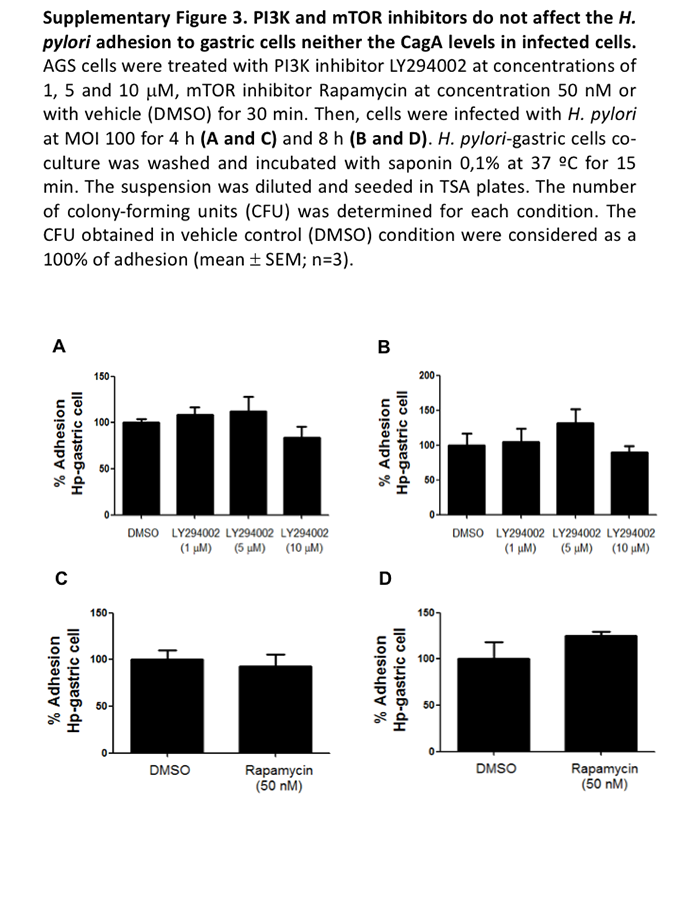

Supplement: Supplementary file 3 [file Image3.TIFF]

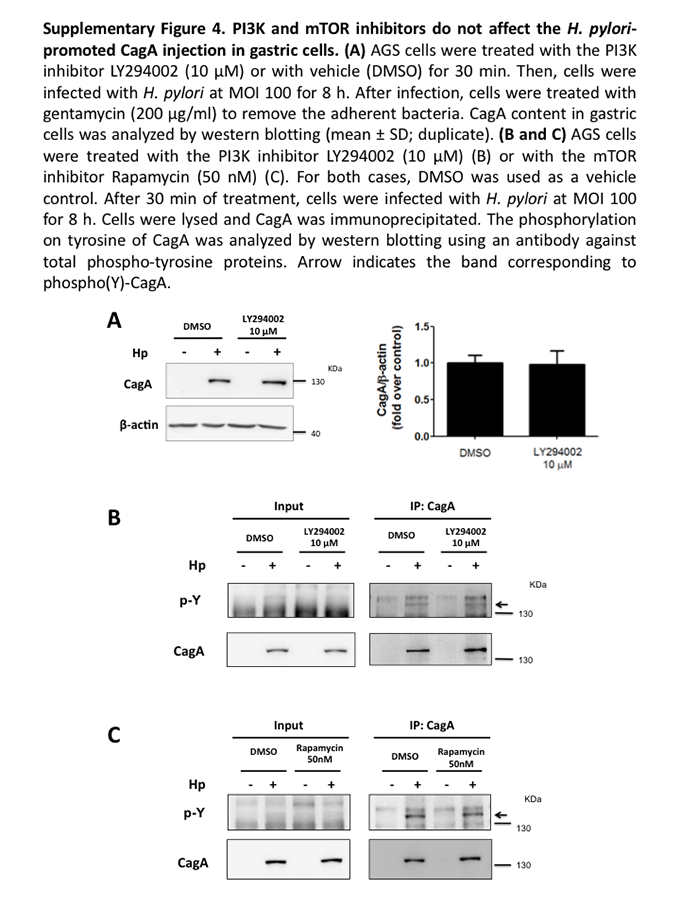

Supplement: Supplementary file 4 [file Image4.TIFF]

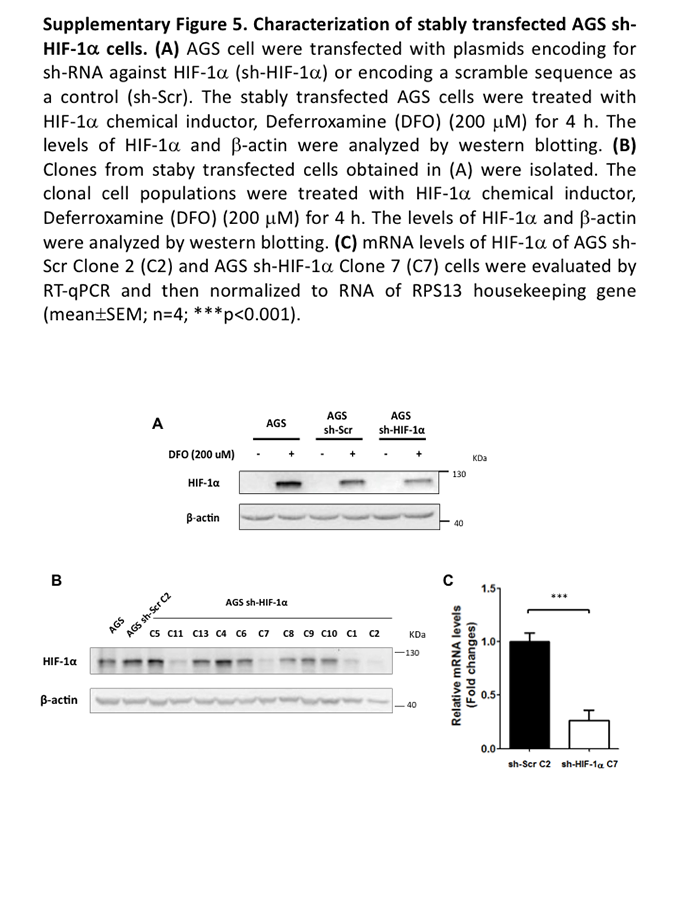

Supplement: Supplementary file 5 [file Image5.TIFF]

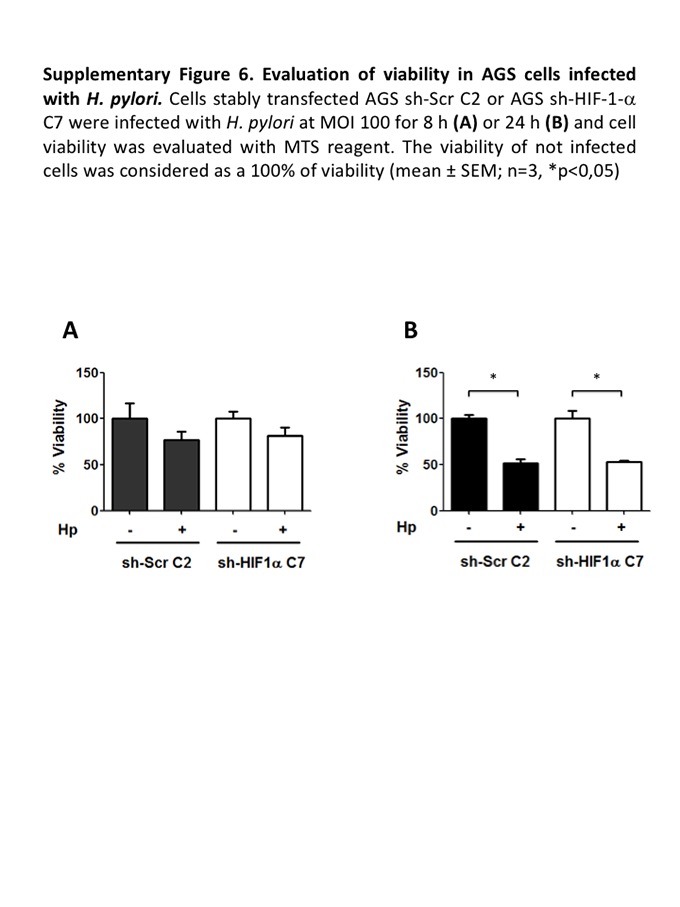

Supplement: Supplementary file 6 [file Image6.TIFF]

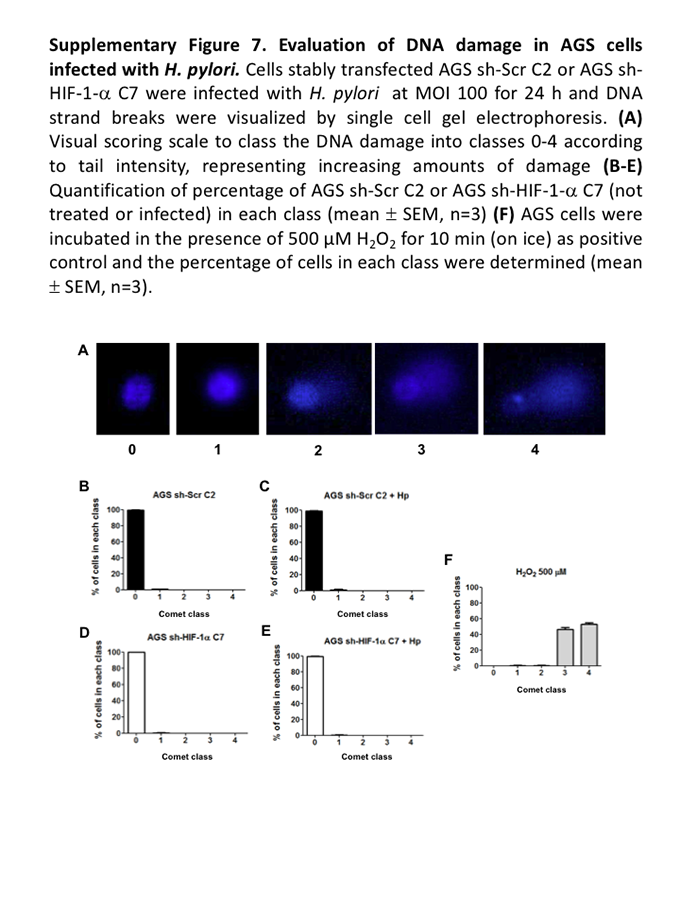

Supplement: Supplementary file 7 [file Image7.TIFF]
