# Supplementary material for: Helicobacter pylori Induced Phosphatidylinositol-3-OH Kinase/mTOR Activation Increases Hypoxia Inducible Factor-1α to Promote Loss of Cyclin D1 and G0/G1 Cell Cycle Arrest in Human Gastric Cells
Source: Front Cell Infect Microbiol. 2017 Mar 28;7:92. doi: 10.3389/fcimb.2017.00092 (PMC5368181; doi:10.3389/fcimb.2017.00092)
Supplement: Supplementary file 8 [file DataSheet1.DOC]

**Supplementary Material**

**Immunofluorescence analysis**

AGS cells were seeded on coverslips and were infected as was described by Valenzuela et al. (2013). After infection, cells were washed with PBS, fixed with Trevors solution, permeabilized with 0.1% Triton X-100 in PBS and blocked with 3% BSA in PBS. After that, cells were probed with a mouse anti-HIF-1α monoclonal antibody (BD #610958, dilution 1:150), Bound antibody was detected with Cy3TM-conjugated anti-mouse antibody (Jackson ImmunoResearch Laboratories #115-165-068, dilution 1:200). Nuclei were stained with DAPI (0,4 μg/ml). The samples were visualized by confocal microscopy (Zeiss LSM-5, Pascal 5 Axiovert 200 microscope) using LSM 5 3.2 image capture and analysis software and a Plan-Apochromat 63× 1.4 NA oil DIC objective. The nuclear area was defined as Region of Interest (ROI) and there the fluorescence intensity of HIF-1α was determined using the ImageJ (NIH) software. Then, the Fluorescence intensity per nuclear area and the percentage of nucleus with positive HIF-1α were calculated.

**Determination of *H. pylori* adhesion to AGS cells.**

AGS cells were seed in a 24-wells culture plate at a density of 1,5 X 10^5^ cells per well. Cells were cultivated for 24 h in RPMI 1640 medium supplemented with 10% FBS and antibiotics. Prior to the infection, culture media was replaced with RPMI without antibiotics and then the PI3K inhibitor LY294002 or mTOR inhibitor Rapamycin were added at concentrations of 1.0, 5.0 and 10.0 μM or 50 nM, respectively, using DMSO as vehicle control. Next, cells were infected at MOI 100 for 4 or 8 h. After the indicated periods of infection, co-cultures were washed with warm RPMI three times to remove the non-adherent bacteria and incubated with 1 ml of saponin 0,1% at 37 ºC for 15 min to lyse the adherent gastric cells. Then lysates were resuspended, diluted and seeded in TSA plates. The number of colony forming units (CFU) was determined.

**Determination of intracellular CagA content in infected AGS cells treated with PI3K inhibitor**

Prior to the infection, culture media was replaced with RPMI without antibiotics and then the PI3K inhibitor LY294002 was added at concentration of 10µM using DMSO as vehicle control. Then, AGS cells were infected at MOI 100 for 8 h. After infection, co-culture was washed with warm RPMI for five times for remove the non-adherent bacteria and incubated with gentamycin (200 µg/ml) at 37°C for 1h to kill extracellular bacteria. After gentamycin treatment, cells were washed for three times with warm RPMI and then total proteins extracts were prepared as was described in Methods section. CagA content was analyzed by western blotting, using for this a mouse anti-CagA monoclonal antibody (Santa Cruz Biotechnology #sc-28368, dilution 1:1000), which was detected with horseradish peroxidase-conjugated anti-mouse antibody (Kirkergaard and Perry Laboratories #214-1806, dilution 1:5000) and the ECL system.

**Determination of tyrosine phosphorylation of CagA in infected AGS cells treated with the PI3K and mTOR inhibitors**

Prior to the infection, culture media was replaced with complete RPMI without antibiotics and then the PI3K inhibitor LY294002 or mTOR inhibitor Rapamycin were added at concentrations of 10 μM and 50 nM, respectively. For both cases, DMSO was used as vehicle control. Then, AGS cells were infected at MOI 100 for 8h. Infected cells were lysed with CHAPS lysis buffer (1% CHAPS, 150 mM NaCl, 30 mM Tris-HCl pH 7.5, 12.5 µg/ml leupeptin, 10 µg/ml antipain, 1 mM benzamide, 1 mM phenylmethylsufonyl fluoride, and 0.5 mM sodium orthovanadate). Lysates were incubated with a mouse anti-CagA monoclonal antibody (Santa Cruz Biotechnology #sc-28368) (2 µg) at 4 °C overnight. Then, a suspension of protein A-coated Sepharose beads (binding capacity 20 mg/ml) was added, and kept in constant rotation for 1 h at 4 °C. After incubation time, beads were pelleted for 1 min at 400 x g and aliquots of supernatants (output) were kept for analysis. The beads were subsequently washed in 1% CHAPS lysis buffer with protease and phosphatase inhibitors, and afterwards samples were resuspended in SDS Sample Buffer (125 mM Tris-HCl, pH 6.8, 2% glycerol, 4% SDS, and 0.05% bromophenol blue). CagA and tyrosine phosphorylated CagA were analyzed in total protein extracts (input) and immunoprecipitates (IPs) and by western blotting using a mouse anti-phospho-tyrosine monoclonal antibody (Santa Cruz Biotechnology #sc-508), which was detected with horseradish peroxidase-conjugated anti-mouse antibody (Kirkergaard and Perry Laboratories #214-1806, dilution 1:5000) and the ECL system.

**Viability determination**

1.0 x 10^4^ cells were seeded in wells of 96-well plates in triplicate for each condition. After 24 h, cells were infected with *H. pylori* at MOI 100 for 8 h or 24 h. Then, cells were washed for three times with PBS and cell viability was measured with CellTiter 96® Aqueous One Solution Cell Proliferation Assay (MTS) kit (Promega, #G5421) following the manufacturer instructions.

**Comet assay**

AGS cells were trypsinized and resuspended in phosphate-buffered saline (PBS) at 10^6^ cells/ml. Thirty microliters of cell suspension was mixed with 140 µL of 1% low-melting point agarose, and two drops of 70 µL of this mixture were placed on a microscope slide pre-coated with 1% normal melting point agarose. A cover slip was put on top of each drop and the gels were allowed to set for 5 min at 4 ºC. Then the cover slip was removed and the slides were immersed in lysis solution (2.5 M NaCl, 0.1 M Na_2_-EDTA, 0.01 M Trizma-BASE pH 10 with 1 % Triton X-100 added prior to use) at 4ºC for 1 h. This procedure removes membranes and most cellular components, including histones, leaving the DNA attached to the nuclear matrix as a nucleoid. The slides were then placed in a horizontal electrophoresis tank filled with freshly prepared alkaline buffer (0.3 M NaOH and 0.001 M Na_2_-EDTA, pH > 13), at 4ºC for 40 min, to allow DNA unwinding. Electrophoresis was carried out for 30 min at 25 V (~0.8 V/cm across the gels and ~300 mA). Finally, slides were neutralized in PBS during 10 min, washed two times (5 min each) with deionized water and fixed 96% ethanol for 5 min.

Gels were stained with 35 µL of 1 µg/mL DAPI, covered with a cover slip and coded. Using a fluorescence microscope, 100 comets were visually classified into five categories, 0–4, representing increasing relative tail intensities and the extent of DNA damage.
